# Effectiveness and Safety of Two Consecutive Cycles of Single Embryo Transfer Compared With One Cycle of Double Embryo Transfer: A Systematic Review and Meta-Analysis

**DOI:** 10.3389/fendo.2022.920973

**Published:** 2022-06-30

**Authors:** Yangqin Peng, Shujuan Ma, Liang Hu, Xiaojuan Wang, Yiquan Xiong, Minghong Yao, Jing Tan, Fei Gong

**Affiliations:** ^1^ Reproductive and Genetic Hospital of CITIC-Xiangya, Clinical Research Center for Reproduction and Genetics in Hunan Province, Changsha City, China; ^2^ Chinese Evidence-Based Medicine Center and CREAT Group, West China Hospital, Sichuan University, Chengdu, China

**Keywords:** single embryo transfer, double embryo transfer, live birth, multiple live birth, perinatal complication, adverse neonatal outcome

## Abstract

**Objective:**

To date, evidence regarding the effectiveness and safety of two consecutive cycles of single embryo transfer (2SETs) compared with one cycle of double embryo transfer (DET) has been inadequate, particularly considering infertile women with different prognostic factors. This study aimed to comprehensively summarize the evidence by comparing 2SETs with DET.

**Methods:**

PubMed, Embase, Cochrane Library databases, ClinicalTrails.gov, and the WHO International Clinical Trials Registry Platform were searched up to March 22, 2022. Peer-reviewed, English-language randomized controlled trials (RCTs) and observational studies (OS) comparing the outcomes of 2SETs with DET in infertile women with their own oocytes and embryos were included. Two authors independently conducted study selection, data extraction, and bias assessment. The Mantel–Haenszel random-effects model was used for pooling RCTs, and a Bayesian design-adjusted model was conducted to synthesize the results from both RCTs and OS.

**Main Results:**

Twelve studies were finally included. Compared with the DET, 2SETs were associated with a similar cumulative live birth rate (LBR; 48.24% vs. 48.91%; OR, 0.97; 95% credible interval (CrI), 0.89–1.13, τ^2 =^ 0.1796; four RCTs and six observational studies; 197,968 women) and a notable lower cumulative multiple birth rate (MBR; 0.87% vs. 17.72%; OR, 0.05; 95% CrI, 0.02–0.10, τ^2 =^ 0.1036; four RCTs and five observational studies; 197,804 women). Subgroup analyses revealed a significant increase in cumulative LBR (OR, 1.33; 95% CrI, 1.29–1.38, τ^2 =^ 0) after two consecutive cycles of single blastocyst transfer compared with one cycle of double blastocyst transfer. Moreover, a lower risk of cesarean section, antepartum hemorrhage, preterm birth, low birth weight, and neonatal intensive care unit admission but a higher gestational age at birth and birth weight were found in the 2SETs group.

**Conclusion:**

Compared to the DET strategy, 2SETs result in a similar LBR while simultaneously reducing the MBR and improving maternal and neonatal adverse outcomes. The 2SETs strategy appears to be especially beneficial for women aged ≤35 years and for blastocyst transfers.

## Introduction

There has been a progressive trend worldwide toward fewer embryo transfers since elective single embryo transfer (eSET) was introduced in 2009 (1, 2). The American Society for Reproductive Medicine data from 2000 to 2017 showed that the proportion of SET increased from 5.7% to 64.2%, and percentage of multiple births among assisted reproduction technology (ART)-conceived infants decreased from 53.1% to 26.4% along with a smooth decrease in preterm birth and low birthweight rates (3). However, results from randomized controlled trials (RCTs), meta-analyses, and statistical modeling of large ART cycle datasets showed that the probability of live birth was reduced with SET compared to double embryo transfers (DETs) (4–7). These studies suggested that although SET may reduce multiple pregnancies, which are highly correlated with maternal and perinatal complications, the risk of endangering the overall live birth rate (LBR) must be considered.

Evidence from the latest Cochrane meta-analysis (8) indicates that compared with one cycle of DET, two consecutive cycles of SET (2SETs) would be an acceptable transplantation strategy to acquire a comparable cumulative LBR while simultaneously reducing multiple pregnancies. However, only four efficacy outcomes were evaluated, and possible antenatal and obstetric complications were not involved in this meta-analysis, the efficacy and safety of 2SETs compared with one cycle of DET need to be assessed more comprehensively. Additionally, existing systematic reviews (4–6, 8) are only based on RCTs (9–12), which mainly enrolled homogeneous populations with limited sample sizes. Given that ART is complicated in clinical practice by differences in age, protocol, cycle, embryo stage, embryo quality, etc., studies with larger sample sizes are needed to refine the population applicability of 2SETs and one cycle of DET (13). Observational studies provide an opportunity to answer these questions (14–16).

We thus conducted a systematic review by integrating information extracted from available RCTs and observational studies to assess the overall effectiveness (i.e., LBR and MBR) of 2SETs versus one cycle of DET in infertile women and also (i) to assess adverse antenatal and neonatal outcomes as comprehensively as possible and (ii) to identify subpopulations that would more clearly benefit from 2SETs vs. DET, considering embryo stage, cycle type, and maternal age.

## Methods

This systematic review adhered to the Preferred Reporting Items for Systematic Reviews and Meta-analyses (PISRM) guidelines (17) and was prospectively registered on PROSPERO (registration ID: CRD42021258452). Institutional review board approval was not required, since this was a meta-analysis of the current literature.

### Eligibility Criteria

RCTs and observational studies comparing the reproductive, obstetric, and perinatal outcomes of two cycles of SET with one cycle of DET in infertile couples with their own oocytes and embryos were included, irrespective of the type of ovarian stimulation protocol, fertilization, or the type and dose of luteal phase support. The 2SETs versus one cycle of DET studies included the following three cycle types: (i) one fresh SET and one subsequent frozen SET versus one fresh cycle of DET, (ii) two consecutive fresh SETs versus one fresh cycle of DET, and (iii) two consecutive frozen SETs versus one frozen cycle of DET. Studies were excluded if there was a disparity between the number of women and the number of cycles.

### Units of Analysis

The primary analysis was per woman; however, we included per pregnancy data for the outcome “miscarriage.” We counted multiple live births (for example, twins or triplets) as one live birth event.

### Literature Search

A systematic electronic literature search of the PubMed, Embase, and Cochrane Library databases and RCT registries (ClinicalTrails.gov and the WHO International Clinical Trials Registry Platform) was conducted from inception through March 22, 2022. The bibliographies of relevant studies and reviews were then scrutinized for any additional eligible studies. For the literature search, terms and descriptors related to human embryo transplantation were combined, and only English language studies were included (see **Supplemental File** for full literature search). Conference abstracts and comments were not considered.

### Study Selection

Citations were merged in the Microsoft Access Database to facilitate management. Duplicates were removed, and two reviewers independently applied the inclusion criteria to all retrieved citations in an un-blinded standardized manner, screened by title, abstract, and full text successively. Any discrepancies were resolved through discussion, and, if necessary, a consensus was reached with the help from the senior authors.

### Outcome Measures

The primary outcomes were LBR, defined as the number of deliveries that resulted in at least one live born baby per cycle, and MBR, defined as a single delivery with more than one newborn per transfer cycle.

The secondary outcomes were maternal pregnancy and neonatal outcomes and complications. These included clinical pregnancy rate (CPR; defined as pregnancy diagnosed by ultrasonographic intrauterine visualization or definitive clinical signs of pregnancy per transfer cycle), multiple pregnancy rate (MPR; defined as a clinical pregnancy with more than one intrauterine fetus per transfer cycle), ectopic pregnancy rate (defined as a pregnancy outside the uterine cavity, diagnosed by ultrasound, surgical visualization, or histopathology per clinical pregnancy cycle), miscarriage rate (defined as the spontaneous loss of a clinical pregnancy occurring before 20 completed weeks of gestation), birth weight, and gestational age at delivery.

Late pregnancy and neonatal complications included gestational diabetes (GDM), pre-eclampsia (PE), antepartum hemorrhage (APH), cesarean section, Apgar 1 < 7, Apgar 5 < 7, neonatal intensive care unit (NICU) admission, and birth defects, and preterm birth rate (defined as a birth after 22 but before 37 completed weeks of gestational age per live birth cycle), very preterm birth rate (defined as a birth after 22 but before 32/34 completed weeks of gestational age per live birth cycle), extremely preterm birth rate (defined as a birth after 22 but before 28 completed weeks of gestational age per live birth cycle), low birth weight rate (defined as the number of babies with birthweight <2,500 g divided by the total number of live birth babies), very low birth weight rate (defined as the number of babies with birthweight < 1,500 g divided by the total number of live birth babies), extremely low birth weight rate (defined as the number of babies with birthweight <1,000 g divided by the total number of live birth babies), and perinatal mortality rate (defined as the number of perinatal deaths divided by the total number of fetuses, including stillbirths and live births).

All the above calculated rates were cumulative incidence rates. The denominators for cumulative CPR, MPR, LBR, and MBR were the number of participants in the earlier of the two consecutive cycles of single embryo transfer.

### Data Extraction

Data on study characteristics (first author, publication year, location, study design, and study period), population (number of participants, age, and major inclusion and exclusion criteria), cycle [type of cycle, first cycle (yes/no), and embryo stage], comparison categories, and clinical outcomes (sample size, number of events, total number, means, standard deviations, risk estimates, 95% CIs, adjusted factors, and conclusions) were extracted onto a piloted structured form independently by two reviewers (YP and SM). The most comprehensive report was given precedence if there were multiple publications from the same study or data source, while the others were potentially used as **Supplementary Information**. When studies had multiple comparisons, only the information and data of interest were extracted. Any uncertainty or disagreements were resolved by discussion, referring back to the original literature.

### Quality Assessment

The quality of the included RCTs was assessed using the Cochrane risk of bias tool (18) based on random sequence generation, allocation concealment, blinding of participants and providers, blinding of outcome assessors, completeness of outcome data, selective outcome reporting, and other potential sources of bias. Each quality item was graded as low risk, high risk, or unclear risk. We defined other biases as trials for which baseline characteristics were not similar between comparison groups, those without *a priori* sample size estimations, and those without an intention to treat analysis. Additionally, the Newcastle–Ottawa quality assessment scale (NOS) (19) was used to assess the quality of the included observational studies. Briefly, this system evaluates studies based on three categories: participant selection (four stars), comparability of study groups (two stars), and assessment of outcome or exposure (three stars). Studies are graded on an ordinal star scoring scale with higher scores representing higher quality.

### Statistical Analysis

Mantel–Haenszel random-effects model was used for pooling RCTs. The Bayesian design-adjusted model has been conducted to synthesize the results from both RCTs and OS, in order to reduce the impact of observational studies’ bias on the combined results (20, 21). To assess the possible impact of patient and embryo characteristics on outcomes, subgroup analyses were pre-specified so that information on the distinct type of study design (RCT or observational studies), the cycle (one fresh SET and one subsequent frozen SET versus one fresh DET, two consecutive fresh SETs versus one fresh DET, or two consecutive frozen SETs versus one frozen DET), embryo stage (cleavage or blastocyst), and maternal age were extracted separately. Maternal age was divided into two categories, with 35 years as the cutoff (≤35 and >35 years). Moreover, sensitivity analyses restricted to the first cycle or eSET cycle were performed to assess the robustness of the findings. Most data were dichotomous; therefore, we used the number of events in each study group to calculate the ORs with 95% confidence interval (CI) or credible interval (CrI). For continuous parameters, data conversion was conducted for all units prior to analysis, and the weighted mean difference (WMD) with 95% CI or CrI were pooled to determine the effect size (22). Heterogeneity was quantified using the estimated tau (2) statistic. Publication bias was assessed using Begg’s test for analyses enrolling more than 10 studies, with p < 0.10 indicating publication bias (23), and the trim-and-fill method was performed if the publication bias was significant. The leave-one-out method was used to evaluate whether any single study dominated the findings. All the statistical analyses were performed using R software, version 4.1.3.

## Results

### Description of Included Studies

**Figure 1:**
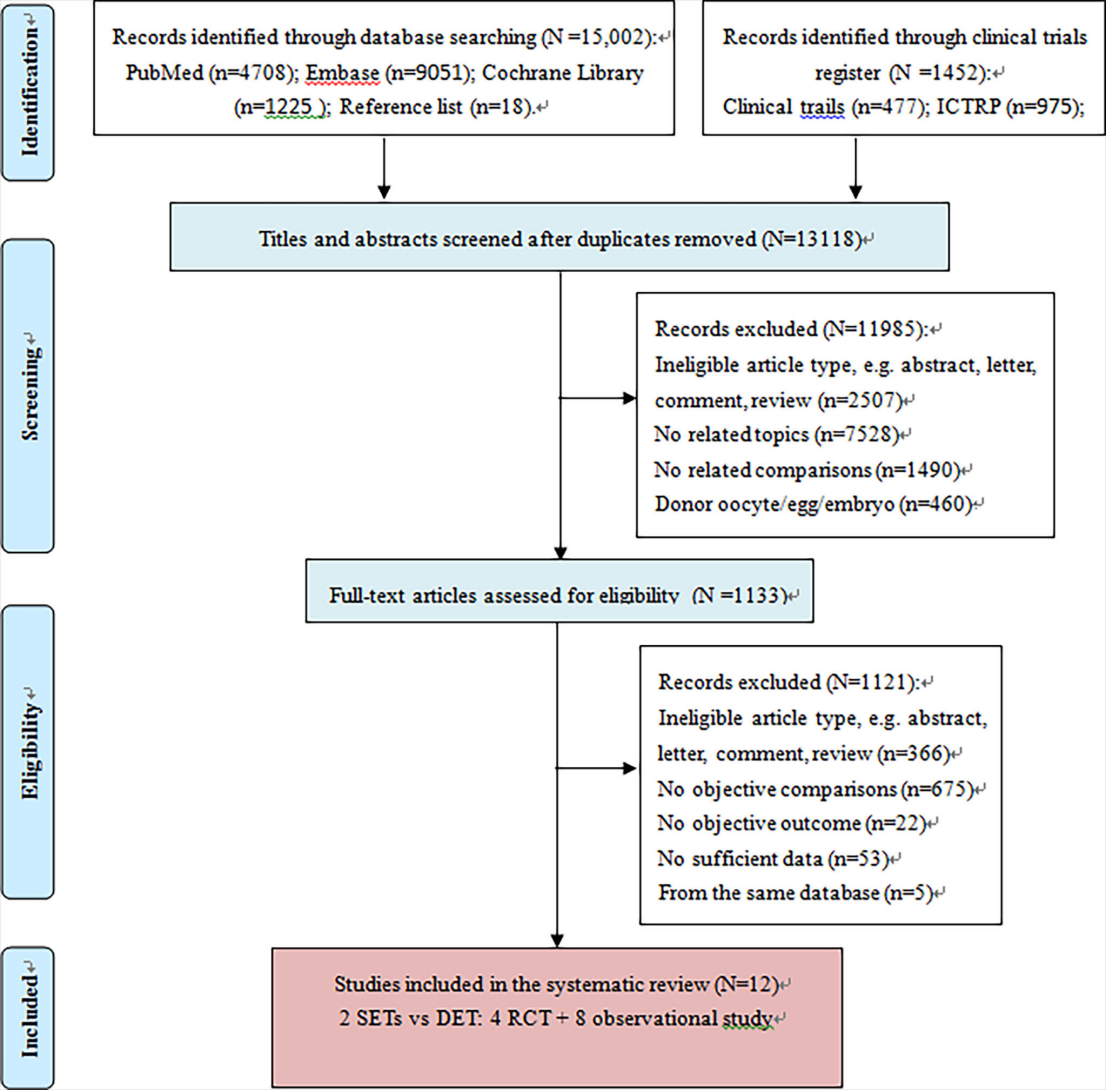
Flow diagram of study selection. DET, double embryo transfer; ICTRP, International Clinical Trials Registry Platform; SET, single embryo transfer; 2SETs, two consecutive cycle of SET; RCT, randomized controlled trial.

The literature search retrieved 16,454 citations. After removing duplicates, 13,118 abstracts were reviewed, and 1,133 full-text articles were further assessed for eligibility. Finally, 12 articles (9–12, 24–32) that offered extractable data for the quantitative meta-analysis were included (**Figure 1**). A total of four RCTs (9–12) and eight observational studies were included (25–32). One of the RCTs (9) separately reported reproduction outcomes and perinatal outcomes in two articles; thus, data were extracted from these two references for this RCT (9, 24). Characteristics of the included studies are presented in **Supplementary Table S1**. The study sample sizes ranged from 42 to 181,523, for a total of 198,892 participants, comprising a total of 40,709 women undergoing two consecutive SET and 158,183 undergoing one DET. Study participants were mainly from the USA (27, 30, 31) (195,892 patients), but were also from Europe (9–12, 25, 26, 29) (2,151 patients) and Asia (28, 32) (849 patients). The women in five studies (25, 26, 28, 30, 31) were recruited during their first cycle, and nine studies (9, 11, 12, 25, 27–30, 32) provided data on eSET. Most studies clarified the type of cycles (10 studies (9, 11, 12, 25–28, 30–32) used one fresh SET and one subsequent frozen SET versus one fresh DET, one study (10) used two consecutive fresh SETs versus one fresh DET, and one study (29) used two consecutive frozen SETs versus one frozen DET) and the stage of embryo transfer [five used cleavage (10, 12, 25, 28, 29), three used blastocysts (26, 27, 32), and four used both (9, 11, 30, 31)]. Ten studies (9–12, 25–27, 29–31) mentioned the age of the recruited infertile women.

The risk of bias assessment of the included RCTs and NOS scores for the observational studies are presented in **Supplementary Table S2**. All RCTs reported the randomization method (9–12), and two (9, 10) conducted blinding for participants or personnel. Two observational studies (25, 26) were prospective cohort studies, while the other six (27–32) were retrospective cohort or database studies. Four (26, 30–32) of the observational studies were awarded seven stars in quality assessment, while the other four (25, 27–29) received eight stars.

### Primary Outcomes

#### Cumulative LBR

**Figure 2:**
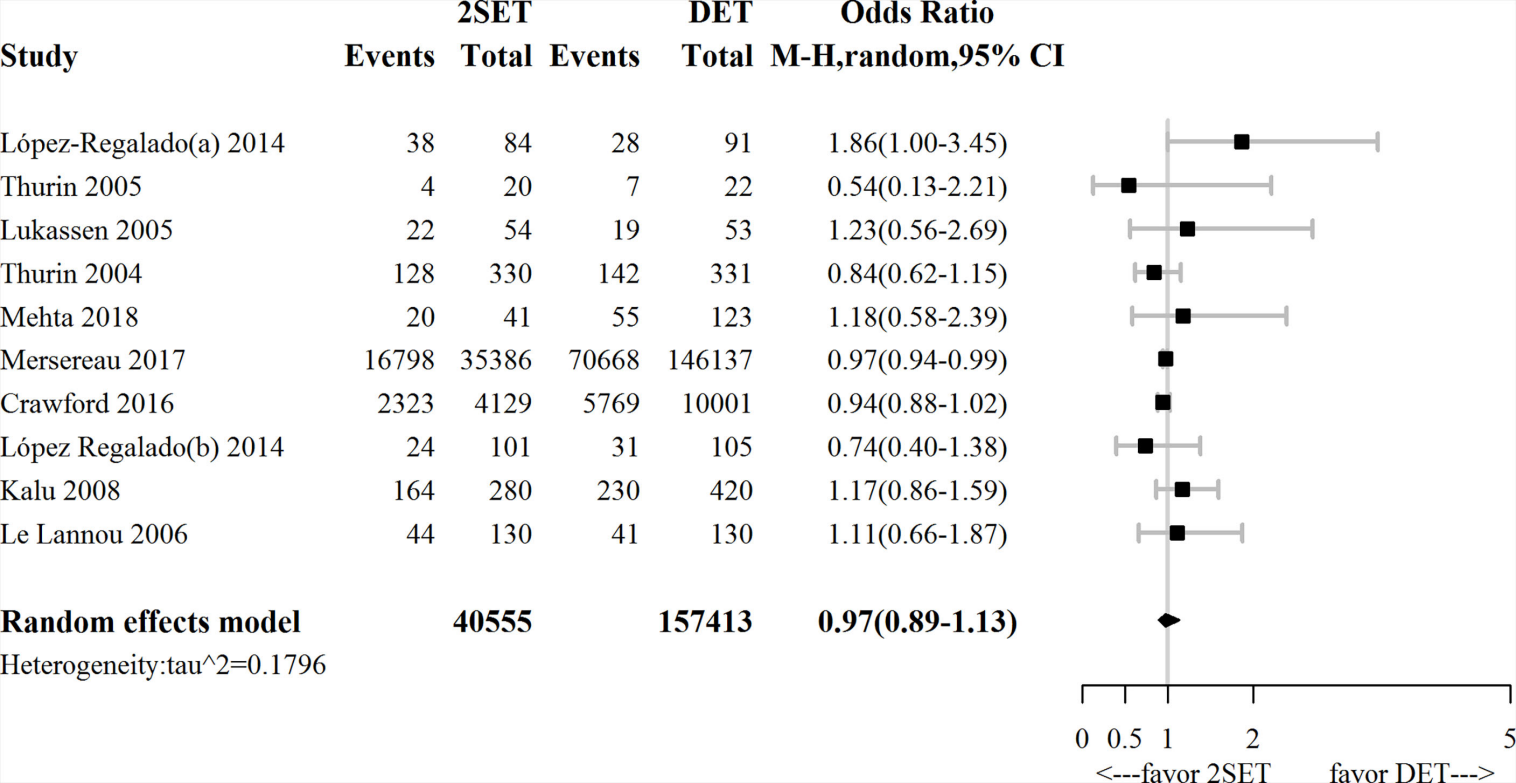
Forest plot comparing cumulative live birth rate after two consecutive cycle of single embryo transfer (2SETs) and one cycle of double embryo transfer (DET).

Ten studies (four RCTs (9–12) and six observational studies (25, 26, 29–32)), which included 197,968 participants, reported cumulative LBR following two consecutive SETs versus one DET. The overall OR for cumulative LBR was 0.97 (95% CrI, 0.89–1.13; τ^2^= 0.1796), indicating a non-significant difference between groups (**Figure 2** and **Table 1**).

**Table 1:** Sensitivity and subgroup analyses comparing cumulative live birth rate after two consecutive cycles of SET (2SETs) and one cycle of DET.

	Studies no.	SET total	DET total	*tau^2^*	OR (95% CI/CrI)
Overall	10	40,555	157413	0.1796	0.97 (0.89–1.13)
Subgroup analyses					
Design					
RCT	4	488	497	0.1069	1.09 (0.68–1.73)
Observational study	6	40,067	156,916	0	0.96 (0.94–0.99)
Cycle					
Fresh+Fresh	1	54	53	0	1.23 (0.56–2.69)
Fresh+Frozen	8	40,400	157,255	0.2182	0.97 (0.87–1.14)
Frozen+Frozen	1	101	105	0	0.74 (0.40–1.38)
Embryo stage					
Cleavage	5	15,499	71,558	0.1496	1.28 (0.61–2.15)
Blastocyst	3	20,577	75,501	0	1.33 (1.29–1.38)
Blastocyst+Cleavage	3	4,479	10,354	0.0609	0.80 (0.47–1.14)
Age (cutoff=35)					
≤35	3	4,513	10,385	0.0915	0.94 (0.80,1.14)
>35	2	71	171	0.0535	0.53 (0.21–1.34)
Sensitivity analyses					
First cycle	4	39,925	156,688	0	0.96 (0.94–0.99)
eSET	7	4,835	10,803	0.2325	0.95 (0.77–1.28)

CI, confidence interval; CrI, credible interval; DET, double embryo transfer; eSET, elective single embryo transfer; OR, odds ratio; RCT, randomized controlled trial; SET, single embryo transfer.

**Figure 3:**
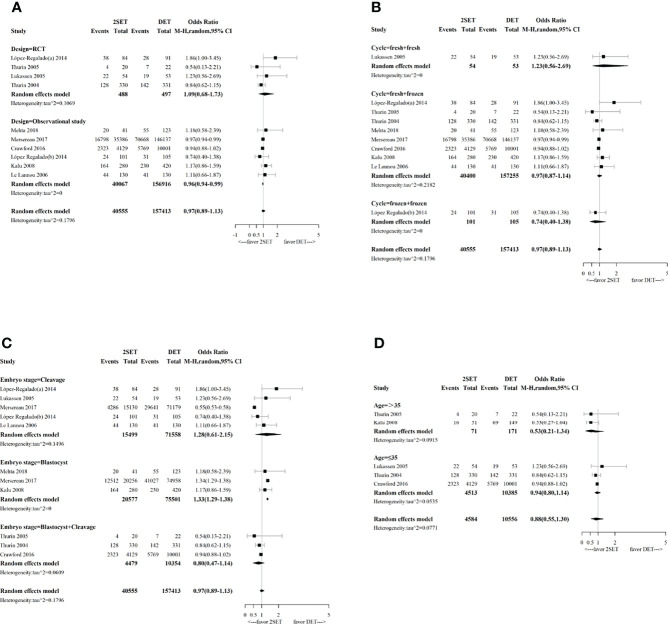
Forest plot with subgroup analysis comparing cumulative live birth rate after two consecutive cycle of single embryo transfer (2SETs) and one cycle of double embryo transfer (DET) based on **(A)** study design, **(B)** cycle type, **(C)** embryo stage and **(D)** maternal age stratification.

Our subgroup analysis concerning study design indicated that the combination of observational studies resulted in a slightly lower OR (0.96; 95% CI, 0.94–0.99; τ^2^= 0), while no change in the OR was observed for the RCTs (OR, 1.09; 95% CI, 0.68–1.73; τ^2^= 0.1069, **Figure 3**).

Subgroup analyses performed according to the type of cycle (two consecutive frozen SETs versus one frozen DET, one fresh SET, and one subsequent frozen SET versus one fresh DET, or two consecutive fresh SETs versus one fresh DET) showed no significant differences (**Figure 3**). Regarding the embryo stage (cleavage and blastocyst), the cumulative LBR was significantly higher after two consecutive cycles of a single blastocyst transfer compared with one cycle of a double blastocyst transfer (OR, 1.33; 95% CI, 1.29–1.38; τ^2^= 0; n=96078; three studies (26, 31, 32), **Figure 3**). However, no differences were noted between two consecutive cycles of a single cleavage embryo transfer and one cycle of a double cleavage embryo transfer (OR, 1.28; 95% CrI, 0.61–2.15; τ^2^= 0.1496; n=87,057; five studies (10, 12, 25, 29, 31), **Figure 3**). Similarly, no differences were noted between two consecutive SET cycles containing a single cleavage embryo transfer and a single blastocyst transfer compared to one DET cycle containing a double cleavage embryo transfer or a double blastocyst transfer (OR, 0.80; 95% CrI, 0.47–1.14; τ^2^= 0.0609; n=14,833; three studies (9, 11, 30), **Figure 3**). For the age group sub-analyses, no differences for cumulative LBR between 2SETs and DET groups were observed for patients aged >35 years or aged ≤35 years (OR, 0.53; 95% CrI, 0.21–1.34; τ^2^= 0.0915; n=242, two studies (11, 26), and OR, 0.94; 95% CrI, 0.80–1.14; τ^2^= 0.0535; n=14,898, three studies (9, 10, 30), respectively, **Figure 3**).

#### Cumulative MBR

**Figure 4:**
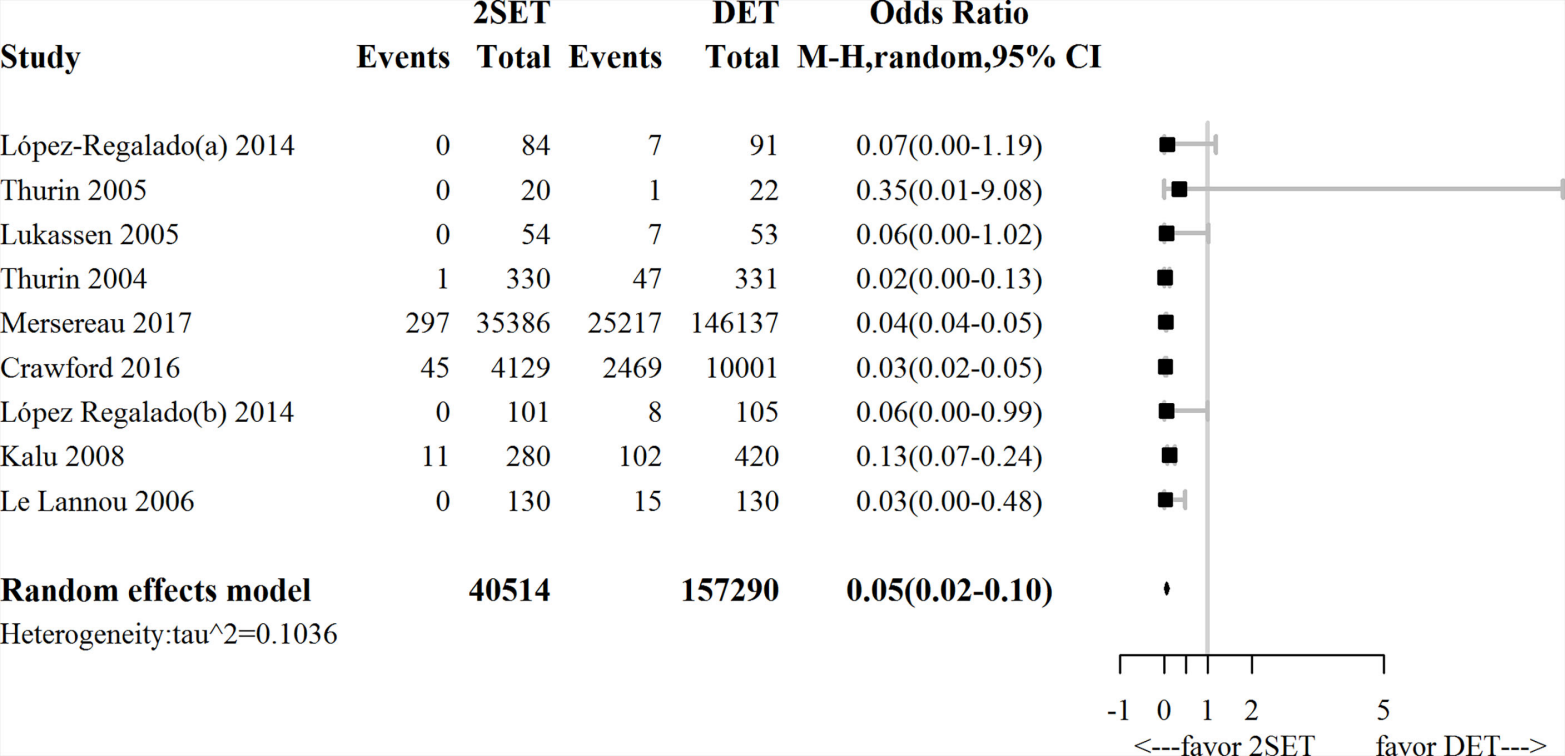
Forest-plot comparing cumulative multiple live birth rate after two consecutive cycle of single embryo transfer (2SETs) and one cycle of double embryo transfer (DET).

For the nine studies that reported cumulative MBR [four RCTs (9–12) and five observational studies (25, 26, 29–31)], which included 197,804 participants, the overall risk of cumulative multiple live births was significantly lower in the 2SETs group than in the DET group (OR, 0.05; 95% CrI, 0.02–0.10; τ^2^= 0.1036) (**Figure 4** and **Table 2**).

**Table 2:** Sensitivity and subgroup analyses comparing cumulative mutiple live birth rate after two consecutive cycles of SET (2SETs) and one cycle of DET.

	Studies no.	SET total	DET total	*tau^2^*	OR (95% CI/CrI)
Overall	9	40,514	157,290	0.1036	0.05 (0.02–0.10)
Subgroup analyses					
Design					
RCT	4	488	497	0	0.05 (0.01–0.18)
Observational study	5	40,026	156,793	0.1052	0.05 (0.03–0.07)
Cycle					
Fresh+Fresh	1	54	53	0	0.06 (0.00–1.02)
Fresh+Frozen	7	40,359	157,132	0.1045	0.05 (0.02–0.11)
Frozen+Frozen	1	101	105	0	0.06 (0.00–0.99)
Embryo stage					
Cleavage	6	15,519	71,580	0.1169	0.04 (0.02–0.13)
Blastocyst	2	20,536	75,378	0.6530	0.07 (0.02–0.22)
Blastocyst+Cleavage	2	4,459	10,332	0.1104	0.03 (0.01–0.07)
Age (cutoff=35)					
≤35	3	4,513	10,385	0.1186	0.03 (0.01–0.07)
>35	2	71	171	0.1119	0.55 (0.03–6.50)
Sensitivity analyses					
First cycle	4	39,925	156,688	0.1164	0.05 (0.03–0.07)
eSET	6	4,794	10,680	0.0923	0.03 (0.02–0.07)

CI, confidence interval; CrI, credible interval; DET, double embryo transfer; eSET, elective single embryo transfer; OR, odds ratio; RCT, randomized controlled trial; SET, single embryo transfer.

**Figure 5:**
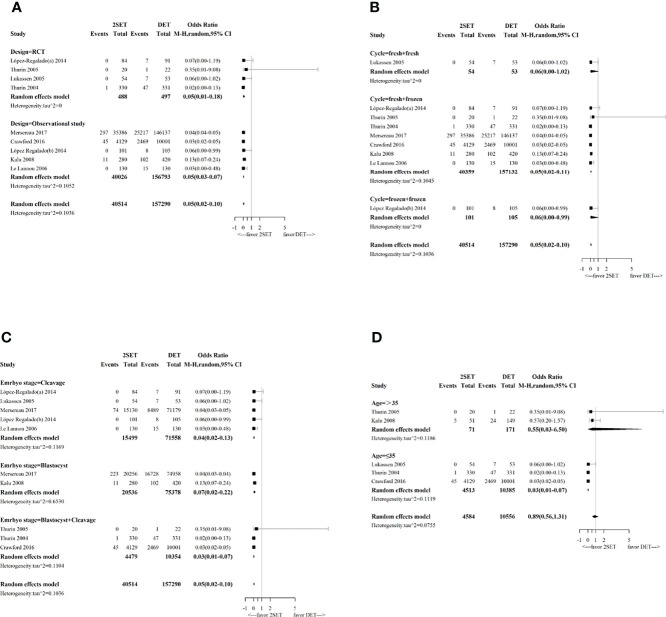
Forest-plot with subgroup analysis comparing cumulative multiple live birth rate after two consecutive cycle of single embryo transfer (2SETs) and one cycle of double embryo transfer (DET) based on: **(A)** study design, **(B)** cycle type, **(C)** embryo stage and **(D)** maternal age stratification.

Subgroup analyses suggested that no differences in terms of cumulative MBR was noted for the two consecutive fresh SET cycles versus one fresh DET cycle subgroup (OR, 0.06; 95% CI, 0.00–1.02; n=107; one study (10), **Figure 5**) or the subgroup of patients aged >35 years (OR, 0.55; 95% CrI, 0.03–6.50; τ^2^= 0.1186; n=242; two studies (11, 26), **Figure 5**).

### Secondary Outcomes

#### Maternal Pregnancy and Neonatal Outcomes

**Table 3** summarizes the overall results of the maternal pregnancy and neonatal outcomes analyses.

**Table 3:** Fertility and maternal and neonatal outcomes of two consecutive cycles of two consecutive cycles of SET(2SETs) and one cycle of DET.

Index	Studies no.	2SETs total	DET total	*tau^2^*	OR/WMD (95% CI/CrI)
Cumulative CPR	7	564	1,272	0.0320	1.37 (0.32–1.84)
Cumulative MPR	5	380	1,089	0.0889	0.07 (0.01–0.48)
Cumulative ectopic pregnancy rate	3	212	270	0.1096	0.64 (0.04–9.13)
Cumulative miscarriage rate	6	316	485	0.0559	1.34(0.94–2.27)
Cumulative prenatal mortality rate	3	171	297	0.1128	0.89 (0.03–14.34)
Cumulative gestational age (week)	2	150	177	0.1794	1.21 (0.27–2.16)
Cumulative preterm birth rate (<37 w) ^§^	4	193	290	0.0752	0.31 (0.16–0.60)
Cumulative very preterm birth rate (<32/34 w)	2	149	244	0.1305	0.34 (0.10–1.51)
Cumulative extremely preterm birth rate (<28 w)	1	20	55	0	8.54 (0.33–218.44)
Cumulative birth weight (g)	2	151	223	17478.5	392.75 (164.07–621.42)
Cumulative low birth weight rate (<2,500 g)	3	171	296	0.0895	0.22 (0.10–0.48)
Cumulative very low birth weight rate (<1,500 g)	2	149	270	0.1160	0.54 (0.20–1.75)
Cumulative extremely low birth weight rate (<1,000 g)	1	20	81	0	12.54 (0.49–319.68)
Cumulative cesarean rate	1	128	142	0	0.33 (0.20–0.55)
Cumulative birth defect rate	2	149	270	0.1104	1.70(0.60–5.05)
Cumulative NICU admission rate	1	129	189	0	0.46 (0.27–0.78)
Cumulative Apgar1 <7 rate	1	129	189	0	1.50 (0.58–3.89)
Cumulative Apgar5 <7 rate	1	129	189	0	0.72 (0.21–2.46)
Cumulative macrosomia rate	1	20	81	0	3.98 (0.08–206.42)
Cumulative GDM rate	1	128	142	0	0.22 (0.02–1.87)
Cumulative PE rate	1	128	142	0	0.90 (0.36–2.25)
Cumulative antepartum hemorrhage rate	1	128	142	0	0.52 (0.29–0.94)
Cumulative antenatal complications rate	2	166	180	0.2832	0.36 (0.13–1.02)

^§^Preterm birth rate was calculated as the number of preterm births divided by the total number of live births (multiple gestations included) in one of included studies (Thurin 2004).

2SETs, two consecutive elective single embryo transfer; CI, confidence interval; CrI, Credible interval; CPR, clinical pregnancy rate; DET, double embryo transfer; LBR, live birth rate; MBR, multiple birth rate; MPR, multiple pregnancy rate; GDM, gestational diabetes mellitus; PE, preeclampsia; PIH, pregnancy-induced hypertension; NICU, neonatal intensive care unit; Apgar1, Apgar score on the first minute of birth; Apgar5, Apgar score on the fifth minute of birth; OR, odds ratio; WMD, weighted mean difference.

No differences in cumulative CPR (10, 12, 25, 27–29, 32), cumulative ectopic pregnancy rates (10, 11, 32), or cumulative miscarriage rates (9, 10, 12, 28, 29, 32) were noted in the 2SETs group compared with the DET group. Nevertheless, the cumulative MPR (12, 27–29, 32) after two SETs was significantly lower than that after one cycle of DET (OR, 0.07; 95% CrI, 0.01–0.48; τ^2^= 0.0889).

There were no significant differences between the 2SETs and DET groups with respect to the risk of cumulative GDM (9), PE (9), or the antenatal complication rate (9, 12). Additionally, no differences were noted regarding the cumulative Apgar 1 < 7 (9), Apgar 5 < 7 (9), perinatal mortality (9, 10, 32), macrosomia (32), birth defects (9, 32), very preterm birth (9, 32), extremely preterm birth (32), very low birth weight (9, 32), or extremely low birth weight (32) rates. Moreover, the cumulative preterm birth rate (9, 10, 12, 32) (OR, 0.31; 95% CrI, 0.16–0.60; τ^2^= 0.0752), low birth weight rate (9, 10, 32) (OR, 0.22; 95% CrI, 0.10–0.48; τ^2^= 0.0895), NICU admission rate (9) (OR, 0.46; 95% CI, 0.27–0.78), APH rate (9) (OR, 0.52; 95% CI, 0.29–0.94), and cesarean section rate (9) (OR, 0.33; 95% CI, 0.20–0.55) were significantly lower in the 2SETs group. Meanwhile, two studies (9, 12) provided data on continuous gestational age at birth and birth weight, including 327 and 374 live birth cycles. A significantly longer gestational age at birth and higher birth weight were found in the 2SETs group compared with the DET group (WMD=1.21 weeks; 95% CI, 0.27–2.16; τ^2^= 0.1794 and WMD=392.75 g; 95% CI, 164.07–621.42; τ^2^= 17,478.45).

#### Sensitivity Analyses and Publication Bias

**Figure 6:**
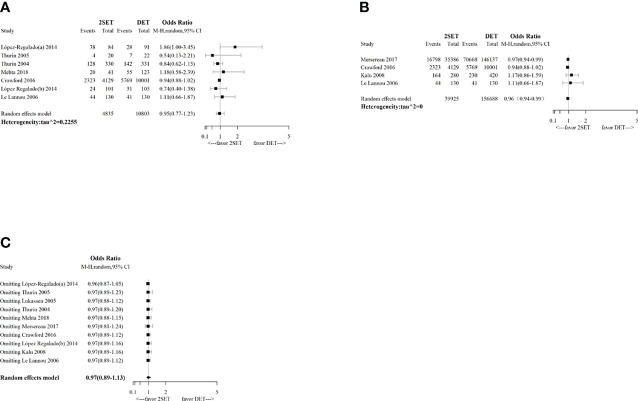
Sensitivity analysis for comparing cumulative live birth rate after two consecutive cycle of single embryo transfer (2SETs) and one cycle of double embryo transfer (DET) based on: **(A)** limited to eSET cycle, **(B)** limited to first cycle, **(C)** leave-one-out method.

**Figure 7:**
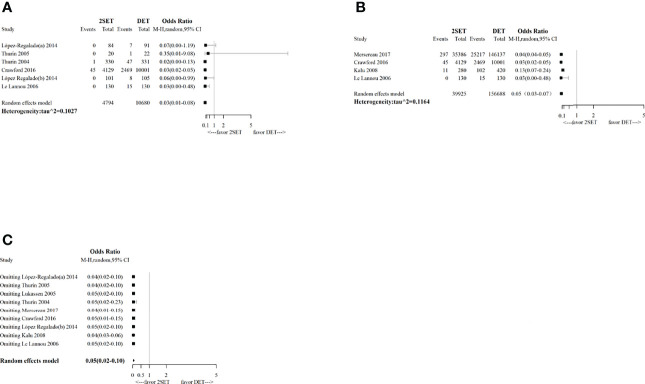
Sensitivity analysis for comparing cumulative multiple live birth rate after two consecutive cycle of single embryo transfer (2SETs) and one cycle of double embryo transfer (DET) based on: **(A)** limited to eSET cycle, **(B)** limited to first cycle, **(C)** leave-one-out method.

The results of the sensitivity analysis conducted using the leave-one-out method showed that the pooled results were robust for cumulative LBR (**Figure 6**), MBR (**Figure 7**), MPR, and CPR; however, the cumulative CPR was significantly higher in the 2SETs group after omitting one study (Lopez-Regalado (b) [2014]). The stability of the overall findings were further confirmed by sensitivity analyses restricted to the first cycle and eSET cycle; however, when restricted to the first cycle, a slightly lower cumulative LBR was found in the 2SETs group (OR, 0.96; 95% CI, 0.94–0.99; τ^2^= 0, **Figure 6**). Publication bias was not assessed due to no more than predetermined 10 studies were included.

## Discussion

### Main Findings

This systematic review showed that two consecutive SETs compared with one DET resulted in a similar probability of cumulative LBR but an enormous decreased risk of cumulative MBR. These findings were confirmed by most sensitivity analyses. For subgroup analyses, cumulative LBR significantly increased after two consecutive cycles of single blastocyst transfer compared with one cycle of double blastocyst transfer. Meanwhile, improved maternal pregnancy and neonatal outcomes were found in the 2SETs group.

### Comparison With Previous Studies

A Cochrane meta-analysis (8) investigated the efficacy of 2SETs versus DET by evaluating four efficacy outcomes, and the pooled overall effects of cumulative LBR were comparable to ours, while safety indicators, such as adverse maternal and neonatal outcomes, were not assessed. Conversely, our meta-analysis included not only more comprehensive reproductive outcome evaluations but also perinatal and neonatal adverse outcome evaluations. Our subgroup analysis based on embryo stage found that the probability of cumulative LBR increased 33% after two consecutive cycles of single blastocyst transfer compared with one cycle of double blastocyst transfer, while only cleavage-stage embryo transfer was included in a previous meta-analysis (8). We also found no difference for cumulative LBR in the subgroup of patients aged both ≤35 and >35 years, which a previous meta-analysis (8) did not mention. Additionally, while the previous meta-analysis (8) included two cycle types (one fresh SET and one subsequent frozen SET vs. one fresh DET and two consecutive fresh SETs vs. one fresh DET), we further supplemented cycle types by adding a third type (two consecutive frozen SETs vs. one frozen DET). However, only one study was included in this subgroup.

### Implications for Clinical Practice and Research

Our findings may be useful for clinical decision making regarding the number of embryos to transfer for different subgroups of infertile women undergoing ART and to encourage future high-quality studies. In summary, the probability of cumulative LBR following 2SETs was about 97% of that achieved by one cycle of DET. However, the risk of cumulative MBR after 2SETs was about 5% that of the DET group, and the risks of cumulative preterm birth and low birth weight were about one-third and one-fifth, respectively.

Interestingly, the direction of the pooled effect size reversed in subgroup comparison after taking the embryo stage into consideration. Our findings suggest that using the 2SETs strategy results in a comparable cumulative live birth regardless of the stage of the embryo that is transferred (even more live births can result when transferred embryos are all blastocysts)s while concurrently decreasing multiple live births. Previous studies (33–35) and a meta-analysis (36) found that blastocyst transfers may increase pregnancy rate, which is particularly relevant in the context of SET aimed to reduce multiple pregnancies.

Another important factor to consider for embryo transfer is maternal age, particularly regarding age-dependent decrements in ovarian function (37). We used 35 years as the cutoff to allow for the inclusion of more primary studies and consistency with previous studies (38, 39). When the age limit was set at ≤35 years, cumulative LBR was not significantly different (0.94, 0.80–1.14) however, cumulative MBR for 2SETs was significantly lower (0.03, 0.01–0.07). When the age was set at >35 years, cumulative LBR in the 2SETs group was still not significantly different (0.53, 0.21–1.34); meanwhile, cumulative MBR was not significantly different (0.55, 0.03–6.50). Our findings thus suggest that younger women (aged ≤35 years) may benefit more from 2SETs, since it has a lower MBR and LBR comparable to that of DET. This might be related to oocyte aneuploidy and decline of uterine receptivity found in older women (7); thus, advanced-age women are more likely to be recommended for multiple embryo transfers (40, 41). Additionally, due to a higher risk of adverse pregnancy outcomes associated with advanced age (42), the safety of 2SETs vs. DET in advanced-age infertile women should also be thoroughly evaluated.

In this study, we also assessed perinatal and neonatal complications following 2SETs and DET. The results showed that both mothers and children conceived through 2SETs had a lower risk of poor outcomes, including lower rates of cesarean section (OR, 0.33), APH (OR, 0.52), preterm birth (OR, 0.31), low birth weight (OR, 0.22), and NICU admissions (OR, 0.46). These findings are consistent with previous studies (43, 44).

Overall, more evidence is needed regarding the number of embryos to transfer. Given the limited number of original studies and small sample size, data were pooled in partial subgroup analyses and perinatal outcomes. Existing national databases and larger well-designed studies are warranted to identify women that would benefit from 2SETs or DET, through a focus on other diverse prognostic profiles, such as recurrent spontaneous abortion and recurrent implantation failure. These studies should also include relatively rare perinatal and neonatal outcomes.

### Strengths and Limitations

This meta-analysis conducted various sensitivity analyses to ensure the robustness of the findings. Additionally, this is the most comprehensive outcome assessment of 2SETs versus DET conducted to date, as it includes many previously unreported adverse outcomes associated with ART treatment, pregnancy, and childbirth, and thus provides more evidence for both healthcare professionals and patients. Furthermore, we included observational studies with diverse populations and larger sample sizes, which allow for conducting subgroup analyses of important prognosticators that have rarely been involved in previous studies, including cycle type, embryo stage, and age stratification it can be useful for accurately identifying subpopulations that would most benefit from each strategy. Meanwhile, we adopt the Bayesian design-adjusted synthesis method for inclusion of results from non-randomized studies to corroborate findings from RCTs. Through this method, the variance of the effects obtained from the non-randomized studies was inflated, so that the weight of the non-randomized studies in the combined results decreased (20, 21).

Several limitations also need to be addressed. First, male factor, ovarian reserve or response, and maternal BMI are other important prognostic factors that we have been focusing on that have significant impacts on the outcomes. Unfortunately, although the baseline distribution of the above indicators were reported in the originally included studies, no further stratified analyses were performed, which limited our secondary analyses. Second, significant heterogeneity was present for some sensitivity and subgroup analyses. Third, the sample size was insufficient for some of the subgroup analyses, maternal pregnancy, and neonatal outcomes, such as the cycle type and the GDM, PE, and NICU admission rates. The relatively small sample size may limit the power, and thus, the capacity of our study to identify a true difference should be interpreted with caution.

## Conclusions

According to the current evidence, the 2SET strategy is associated with a similar LBR and better obstetrics and neonatal outcomes compared with one cycle of DET. Subgroup analyses also showed that some characteristics of the patients and embryos were critical prognostic factors regarding the reproductive outcomes of ART. For example, differences in cumulative LBR between the groups were reversed in patients who underwent two consecutive cycles of a single blastocyst transfer, but non-significant cumulative LBR and significantly lower cumulative MBR were found in the subgroup analyses of patients aged ≤35 years, which suggests that infertile women aged ≤35 years and those receiving blastocyst transfers would benefit more from the two consecutive SET strategy. Further high-quality RCTs or national registry-based cohort studies are needed to confirm these findings and to assess suitable transplantation strategies for infertile women with other prognostic characteristics.

## Data Availability Statement

The original contributions presented in the study are included in the article/**Supplementary Material**. Further inquiries can be directed to the corresponding authors.

## Author Contributions

Study concept and design: YP, SM and JT. Acquisition, analysis, or interpretation of data: YP, SM, XW, YX, MY and JT. Statistical analysis: YP, SM, YX and MY. Drafting of the manuscript: YP and SM. Critical revision of the manuscript for important intellectual content: LH JT and FG. Obtained funding: FG Administrative, technical, or material support: JT and FG. Study supervision: JT and FG. All authors contributed to the article and approved the submitted version.

## Funding

This work was funded by National KeyResearch & Developmental Program of China (2018YFC1004901) and Hunan Provincial Grant for Innovative Province Construction (2019SK4012).

## Conflict of Interest

The authors declare that the research was conducted in the absence of any commercial or financial relationships that could be construed as a potential conflict of interest.

## Publisher’s Note

All claims expressed in this article are solely those of the authors and do not necessarily represent those of their affiliated organizations, or those of the publisher, the editors and the reviewers. Any product that may be evaluated in this article, or claim that may be made by its manufacturer, is not guaranteed or endorsed by the publisher.
