# Supplementary material for: Effectiveness and Safety of Two Consecutive Cycles of Single Embryo Transfer Compared With One Cycle of Double Embryo Transfer: A Systematic Review and Meta-Analysis
Source: Front Endocrinol (Lausanne). 2022 Jun 30;13:920973. doi: 10.3389/fendo.2022.920973 (PMC9279578; doi:10.3389/fendo.2022.920973)
Supplement: Supplementary file 1 [file DataSheet_1.zip › Supplementary Table S1.DOCX]

| **Table S1. The characteristics of the included studies.** | | | | | | | | | | | |
| --- | --- | --- | --- | --- | --- | --- | --- | --- | --- | --- | --- |
| **Study (year)** | **Design** | **Location** | **Study period** | **Participants** | **Cycle** | **First cycle** | **Embryo stage** | **Comparison** | **Total number** | **Age (mean/ median/ range)** | **Clinical outcomes** |
| López-Regalado 2014 (a) | RCT | Spain | 2010.01 - 2012.12 | **Included:** <38 years, BMI 19-29 kg/m^2^, D3 FSH<15mUI/ml, first cycle of IVF/ICSI or second cycle after a prior attempt with a positive pregnancy test result; **Excluded:** ≥ 5 years of infertility, previous surgery, uterine malformations, ≥2 repeated spontaneous abortions. | Fresh/Frozen | First/ Second | Cleavage | eSET | 84 | 32.2 ± 3.6 (21-37) | CPR, MPR, LBR, MBR, abortion rate weeks of gestation at delivery. |
|  |  |  |  |  |  |  |  | DET | 91 | 31.7 ± 3.8 (20-37) |  |
| Thurin 2005 | Multi-center double-blind RCT | Sweden | NA | **Included:** ≥36 years. First or second IVF/ICSI cycle. ≥2 good quality embryos available | Fresh | First/ Second | Cleavage + blastocyst | eSET | 20 | ≥36 | LBR, MBR, cLBR. |
|  |  |  |  |  |  |  |  | DET | 22 | ≥36 |  |
| Lukassen 2005 | Open-label RCT | The Netherlands | 2001.01-2003.02 | **Included:** ﬁrst IVF/ICSI cycle ever or the first cycle after a successful treatment, < 35 years, basal FSH level < 10 IU/l, ≥2 good embryos available for transfer on D3; **Excluded:** with a medical reason for elective SET. | Fresh | First/ Second | Cleavage | SET | 54 | 30.2 ± 3.2 (20-34) | CPR, miscarriage rate, ectopic pregnancy rate, LBR, MBR, perinatal death rate, preterm birth rate, low birthweight rate. |
|  |  |  |  |  |  |  |  | DET | 53 | 31.2 ± 2.9 (25-34) |  |
| Thurin 2004 / Kjellberg 2006 | Multi-center, double-blind RCT | Sweden | 2000.05-2003.10 | **Included:** < 36 years at the time of the transfer of fresh embryo, first or second IVF cycle, had ≥2 embryos of good quality available. | Fresh/Frozen | First/ Second | Cleavage/Blastocyst | eSET | 330 | 30.9 ± 3.0 (22.6–35.9) | Ectopic pregnancy rate, miscarriage rate, ongoing pregnancy rate, stillbirth rate, LBR. |
|  |  |  |  |  |  |  |  | DET | 331 | 30.8 ± 3.0 (21.6–35.9) |  |
| Mehta 2018 | Retro-spective cohort study | India | 2015.01 - 2015.12 | **Included:** having ≥2 transferable blastocysts; **Excluded:** donor oocytes, had an all freeze cycle due to premature progesterone elevation, the risk of OHSS or unfavorable endometrium. | Fresh | NA | Blastocyst | eSBT | 41 | 34.5 ± 4.3 | Pregnancy rate, implantation rate, CPR, MPR, miscarriage rate, ectopic pregnancy, LBR. |
|  |  |  |  |  |  |  |  | DBT | 123 | 35.0 ± 3.4 |  |
| Mersereau 2017 | Retro-spective database analysis | USA | 2004 - 2013 | **Included:** first autologous fresh cycle; **Excluded:** history of prior gonadotropin or IVF treatment, research cycles, embryo banking cycles, or cycles that used a gestational carrier, PGS/PGD cycles. | Fresh | Yes | Cleavage/Blastocyst | SET | 15130 | 33.5 ± 4.2 | LBR, MBR. |
|  |  |  |  |  |  |  |  | SBT | 20256 |  |  |
|  |  |  |  |  |  |  |  | DET | 71179 |  |  |
|  |  |  |  |  |  |  |  | DBT | 74958 |  |  |
| Crawford 2016 | Retro-spective database analysis | USA | 2012 - 2013 | **Included:** <35 years, with no previous ART treatment, fresh, autologous cycles, ≥1 embryo was cryopreserved. | Fresh | Yes | Cleavage/Blastocyst | eSET | 4129 | NA | LBR, MBR. |
|  |  |  |  |  |  |  |  | eDET | 10001 |  |  |
| López Regalado 2014 (b) | Retro-spective cohort study | Spain | 2010.01 - 2013.06 | **Included:** <38 years, BMI 19-29 kg/m^2^, D3 FSH< 15mUI/ml, first cycle IVF/ICSI or second cycle with previous pregnancy not carried to term; **Excluded:** ≥ 5 years of infertility, previous surgery, uterine malformations, repeated miscarriages and previous unsuccessful complete cycles of IVF/ICSI. | Frozen | First/ Second | Cleavage | SET | 101 | 33.5 ± 3.7（21-37） | Implantation rate, miscarriage rate, ongoing pregnancy rate, CPR, MPR, LBR, MBR. |
|  |  |  |  |  |  |  |  | DET | 105 | 34.3 ± 4.1（21-37） |  |
| Nakagawa 2010 | Retro-spective cohort study | Japan | 2005.01-2008.12 | **Included:** first ART treatment, DET was mainly carried out between 2005.01-2008.03, and SET was carried out between 2008.04 -2008.12 | Fresh | Yes | Cleavage | SET | 102 | 34.3 ± 0.4 | CPR, miscarriage rate, ongoing pregnancy rate, LBR, MPR. |
|  |  |  |  |  |  |  |  | DET | 583 | 35.7 ± 0.2 |  |
| Styer 2008 | Retro-spective cohort study | USA | 2002.01-2006.03 | **Included:** fresh blastocyst transfer, ≤37 years, ≥5 embryos at the six- to eight-cell stage on day 3. | Fresh | No | Blastocyst | eSBT | 52 | 31.4 ± 3.6 | Positive serum HCG rate, CPR, LBR, pregnancy loss rate, implantation rate, ectopic pregnancy rate, MPR. |
|  |  |  |  |  |  |  |  | DBT | 187 | 32.0 ± 3.5 |  |
| Kalu 2008 | Pro-spective cohort study | UK | 2005.01-2006.12 | **Included:** 700 cycles of IVF/ICSI involving fresh blastocyst transfer and a total of 102 cycles of FBT performed in women aged 25–43 years. | Fresh | Yes | Blastocyst | SBT | 280 | 25-43 | CPR, MPR, LBR, MBR. |
|  |  |  |  |  |  |  |  | DBT | 420 |  |  |
| Le Lannou 2006 | Pro-spective cohort study | France | 2002.06-2004.12 | **Included:** ﬁrst cycle of treatment, < 38 years, had ≥2 good quality embryos. | Fresh | Yes | Cleavage | SET | 130 | 30.1  (21–37) | LBR, CPR, implantation rate, LBR, MBR, ectopic pregnancy rate, miscarriage rate. |
|  |  |  |  |  |  |  |  | DET | 130 | 31.8  (24–37) |  |
| Note:  Abbreviation: ART, assisted reproductive technology; BMI, body Mass Index; CPR, clinical pregnancy rate; DBT, double blastocyst transfer; DET, double embryo transfer; ET, embryo transfer; FBT, frozen blastocyst transfer; FSH, follicle-stimulating hormone; IVF, in-vitro fertilization; LBR, live birth rate; MBR, multiple live birth rate; MPR, multiple pregnancy rate; NA, not applicable or not reported; OHSS, ovarian hyperstimulation syndrome; PGD, preimplantation genetic diagnosis; PGS, preimplantation genetic screening; SBT, single blastocyst transfer; SET, single embryo transfer. | | | | | | | | | | | |
|  |  |  |  |  |  |  |  |  |  |  |  |
|  |  |  |  |  |  |  |  |  |  |  |  |
|  |  |  |  |  |  |  |  |  |  |  |  |
