# Supplementary material for: Effectiveness and Safety of Two Consecutive Cycles of Single Embryo Transfer Compared With One Cycle of Double Embryo Transfer: A Systematic Review and Meta-Analysis
Source: Front Endocrinol (Lausanne). 2022 Jun 30;13:920973. doi: 10.3389/fendo.2022.920973 (PMC9279578; doi:10.3389/fendo.2022.920973)
Supplement: Supplementary file 1 [file DataSheet_1.zip › Supplementary File_Search Strategy.DOC]

[Search](../../../../D:/info/YoudaoDict_setup_3987/%25E6%259C%2589%25E9%2581%2593%25E8%25AF%258D%25E5%2585%25B8%25E5%25AE%2589%25E8%25A3%2585%25E6%2596%2587%25E4%25BB%25B6/Dict/7.5.1.0/resultui/dict/%3Fkeyword=search) [S](../../../../D:/info/YoudaoDict_setup_3987/%25E6%259C%2589%25E9%2581%2593%25E8%25AF%258D%25E5%2585%25B8%25E5%25AE%2589%25E8%25A3%2585%25E6%2596%2587%25E4%25BB%25B6/Dict/7.5.1.0/resultui/dict/%3Fkeyword=strategy)trategy

(((((((((((((Assisted Reproductive Techn*) OR (assisted conception)) OR (In vitro fertilization*)) OR (In-vitro fertilization*)) OR (Fertilization in Vitro)) OR (IVF)) OR (Intracytoplasmic Sperm Injection)) OR (Intracytoplasmic Sperm transfer)) OR (ICSI)) OR (embryo trans*)) OR (blastocyst trans*)) OR (embryo replace*)) OR (blastocyst replace*)) AND ((((((((((((((((((((((((((((("Single embryo") OR ("single blastocyst")) OR ("one embryo")) OR ("one blastocyst")) OR (Single-embryo)) OR (single-blastocyst)) OR (one-embryo)) OR (one-blastocyst)) OR (SET)) OR (SBT)) OR (eSET)) OR (eSBT)) OR ("double embryo*")) OR ("double blastocyst*")) OR ("2 embryo*")) OR ("2 blastocyst*")) OR ("two embryo*")) OR ("two blastocyst*")) OR (double-embryo*)) OR (double-blastocyst*)) OR (2-embryo*)) OR (2-blastocyst*)) OR (two-embryo*)) OR (two-blastocyst*)) OR (DET)) OR (DBT)) OR (eDET)) OR (eDBT)))
