# Supplementary material for: Effectiveness and Safety of Two Consecutive Cycles of Single Embryo Transfer Compared With One Cycle of Double Embryo Transfer: A Systematic Review and Meta-Analysis
Source: Front Endocrinol (Lausanne). 2022 Jun 30;13:920973. doi: 10.3389/fendo.2022.920973 (PMC9279578; doi:10.3389/fendo.2022.920973)
Supplement: Supplementary file 1 [file DataSheet_1.zip › Supplementary Table S2.DOC]

Table S2 Methodological quality of included studies.

| **RCTs** | **Cochrane risk of bias tool** | | | | | | | | | |
| --- | --- | --- | --- | --- | --- | --- | --- | --- | --- | --- |
|  | Random sequence generation (selection bias) | Allocation concealment (selection bias) | Blinding for participants and personnel (performance bias) | | Blinding of outcome assessment (detection bias) | | Incomplete outcome data (attrition bias) | Selective reporting (reporting bias) | | Other bias |
| López-Regalado, M. L. 2014(a) | low | high | high | | low | | low | low | | low |
| Thurin 2005 | low | unclear | low | | low | | low | low | | unclear |
| Lukassen, H. G. M. 2005 | low | unclear | high | | low | | low | low | | low |
| Thurin, A. 2004/Kjellberg, A. T. 2006 | low | unclear | low | | low | | low | low | | unclear |
|  |  |  |  | |  | |  |  | |  |
| **Observational studies** | **Newcastle-Ottawa Scale (NOS)** | | | | | | | | | |
|  | Selection | | | Comparability | | Outcome | | | Overall quality | |
| Mehta, V. 2018 | 4 | | | 0 | | 3 | | | 7 | |
| Mersereau, J. 2017 | 4 | | | 0 | | 3 | | | 7 | |
| Crawford, S. 2016 | 4 | | | 0 | | 3 | | | 7 | |
| López Regalado, M. L. 2014(b) | 4 | | | 1 | | 3 | | | 8 | |
| Nakagawa, K. 2010 | 4 | | | 1 | | 3 | | | 8 | |
| Styer, A. K. 2008 | 4 | | | 1 | | 3 | | | 8 | |
| Kalu, E. 2008 | 4 | | | 0 | | 3 | | | 7 | |
| Le Lannou, D. 2006 | 4 | | | 1 | | 3 | | | 8 | |
